# Cardiopulmonary Resuscitation-Induced Hardware Failure After Surgical Stabilization of Flail Chest

**DOI:** 10.7759/cureus.15549

**Published:** 2021-06-09

**Authors:** William T Head, Christopher S Thomas, Evert A Eriksson

**Affiliations:** 1 Department of Surgery, Division of Trauma and Acute Care Surgery, Medical University of South Carolina, Charleston, USA

**Keywords:** rib fractures, flail chest, chest wall trauma, hardware failure, surgical stabilization of rib fractures, rib fixation

## Abstract

Flail chest occurs when three or more ribs have concurrent fractures in two or more places. Flail chest is a marker of injury severity and is associated with increased morbidity and mortality. The management of flail chest includes multiple nonoperative components in addition to surgical stabilization, which has been shown to lower mortality rates to those of multiple rib fractures with a stable chest wall (i.e., no flail chest). The resulting stability of the chest wall may be a more accurate prognostic indicator than the actual number of ribs fractured. Surgical stabilization has been associated with various complications. The overall incidence of hardware failure is relatively rare and often involves the anterolateral and lateral regions of the chest wall. We present a unique case of a 48-year-old male involved in a motor vehicle accident with multiple traumatic injuries, including flail chest. He ultimately underwent surgical stabilization across six separate ribs in nine total locations. The patient’s condition deteriorated several weeks later, and he required cardiopulmonary resuscitation. High impact forces caused hardware failure in three separate locations along the chest wall, i.e., anteriorly, anterolaterally, and posterolaterally. The most significant failure occurred anteriorly with sternal plate and screw separation. We suspect that hardware failure in the anterior and anterolateral regions indicates that the sternum and costochondral junction may be dynamic areas of the chest wall that dissipate forces differently than do the bone of ribs.

## Introduction

Rib fractures occur frequently in patients with thoracic trauma and are among the most common injuries encountered after a blunt mechanism of injury [1]. Their presence largely serves as a marker of injury severity, and they have been associated with a 10% overall mortality rate. Moreover, an increased number of fractures has been associated with increased mortality, which is particularly concerning for those with flail chest and those over 65 years old. Flail chest can encompass two separate patient presentations. Clinically, it signifies the paradoxical motion of fractured ribs throughout the respiratory cycle; radiologically, it signifies three or more ribs that have concurrent fractures in two or more places [2]. Flail chest injuries differ in outcome severity when compared to not only single but also multiple rib fracture injuries not meeting the flail chest diagnostic criteria. An analysis of over 100,000 patients with rib fractures showed that flail chest injuries were significantly more likely to require the following: epidural catheter, chest tube, tracheostomy, mechanical ventilation, intensive care unit (ICU) admission, increased length of stay, and hospital readmission [3]. They were also more likely to experience the following complications: pneumonia, acute renal failure, bleeding requiring transfusion, major wound disruption, surgical site infection, and short-term and long-term mortality [3].

## Case presentation

We present a 48-year-old male who arrived at our academic level I trauma center complaining of left shoulder, back, and hip pain with corresponding flail chest. Initial workup identified several injuries, including a left pubic ramus fracture, a left sacral fracture, a left scapular fracture, a chronic right dislocated temporomandibular joint, a left basilar pulmonary contusion, a left hemopneumothorax, a small retroperitoneal hematoma without active extravasation, and flail chest. While flail chest was identified, the initial computerized tomography (CT) report did not identify all the patient’s rib fractures. He was not originally deemed an optimal surgical fixation candidate for several reasons related to the initial imaging: many of the fractures were too close to the corresponding transverse process for fixation, many of the fractures were in line with minimal displacement, and the costocartillage fractures were not readily discernible. Due to the extent of his injuries, the decision was made to admit him to the Surgical Trauma Intensive Care Unit (STICU).

On hospital day (HD) two, orthopedic surgery was consulted and recommended nonoperative treatment of his pelvic and scapular injuries. A thoracic epidural catheter was placed for pain management, and a chest tube was inserted for an enlarging hemothorax. The patient was transferred to a lower level of care but later developed respiratory distress and was readmitted to the STICU on BiPAP. On HD 10, he was intubated for further respiratory insufficiency and altered mental status. A repeat chest CT scan then revealed greater displacement and offset of the previously identified fractures as well as improved visibility of the costocartillage fractures (Table 1). He was then deemed to be an appropriate surgical candidate on HD 13 and received surgical stabilization of rib fractures (SSRF) on the left side. He returned to the operating room on HD 15 for completion of SSRF. MatrixRIB^TM^ plates and sternal locking screws (MatrixRIB^TM^ Fixation System, DePuy Synthes, Raynham, MA) were used across six separate ribs in nine total locations (Table 1). Other fracture locations were not deemed appropriate for fixation due to the patient’s body habitus and concern for damage to surrounding tissue. The procedures were without complications, and he returned to the STICU postoperatively.

**Table 1:** Fracture patterns and primary hardware changes after CPR. Fracture descriptions are in parentheses: Nondisplaced = ≥90% contact between cortical surfaces; minimally offset = <90% but ≥50% contact; moderately offset = <50% but >0% contact; displaced = no cortical contact. SSRF: surgical stabilization of rib fractures; CPR: cardiopulmonary resuscitation

Rib	Fractures before SSRF	Hardware locations	Fractures after SSRF and CPR	Hardware changes
Left 1	Anterior (nondisplaced)		Anterior (nondisplaced)	
Left 2	Anterior (nondisplaced)		Anterior (nondisplaced)	
	Anterolateral (moderately offset)		Anterolateral (moderately offset)	
	Lateral (displaced – 11 mm)		Lateral (moderately offset)	
Right 2	Lateral (nondisplaced)		Lateral (nondisplaced)	
Left 3	Anterior (moderately offset)		Anterior (moderately offset)	
	Anterolateral (displaced – 10 mm)		Anterolateral (moderately offset)	
	Posterolateral (displaced – 21 mm)		Posterolateral (displaced – 18 mm)	
Left 4	Anterior (displaced – 13 mm)		Anterior (moderately offset)	
	Anterolateral (moderately offset)	Anterolateral	Anterolateral (fixated)	Stable
	Posterolateral (displaced – 30 mm)		Posterolateral (displaced – 31 mm)	
Left 5	Anterior (displaced – 13 mm)	Anterior	Anterior (fixated)	Failure
	Anterolateral (moderately offset)	Anterolateral	Anterolateral (fixated)	Failure
	Posterolateral (displaced – 18 mm)		Posterolateral (displaced – 24 mm)	
Left 6	Anterolateral (minimally offset)	Anterolateral	Anterolateral (fixated)	Stable
	Posterolateral (displaced – 18 mm)	Posterolateral	Posterolateral (fixated)	Failure
Left 7	Anterolateral (minimally offset)	Anterolateral	Anterolateral (fixated)	Stable
	Posterolateral (moderately offset)	Posterolateral	Posterolateral (fixated)	Stable
Left 8	Anterolateral (minimally offset)		Anterolateral (healing)	
	Posterolateral (displaced – 16 mm)	Posterolateral	Posterolateral (fixated)	Stable
Left 9	Posterolateral (moderately offset)	Posterolateral	Posterolateral (fixated)	Stable
Left 10	Posterolateral (minimally offset)		Posterolateral (minimally offset)	
Left 11	Posterolateral (minimally offset)		Posterolateral (minimally offset)	

**Figure 1:**
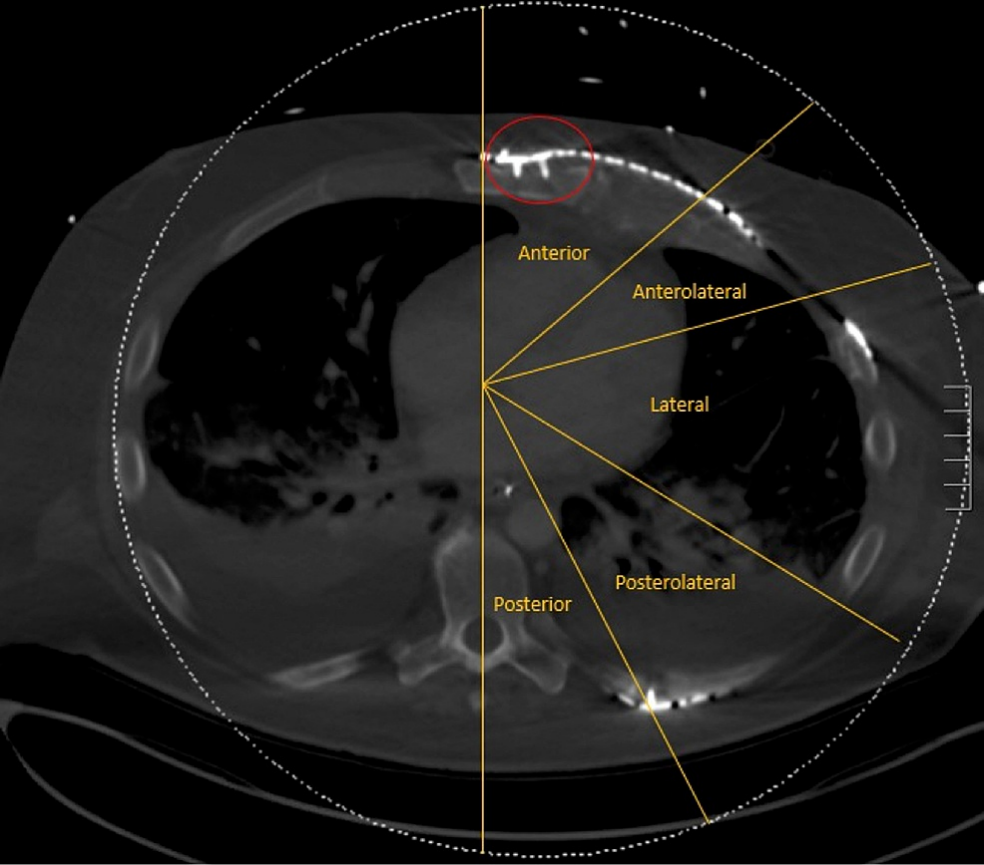
Hardware failure with screw and plate separation. Computerized tomography image highlighting locking screw and plate separation from the sternum at the level of the fifth rib.

He was able to be extubated on HD 16. On HD 40, the patient was reintubated for respiratory insufficiency but afterward became bradycardic with pulseless electrical activity (PEA) and required cardiopulmonary resuscitation (CPR) with the return of spontaneous circulation after two rounds of defibrillation. This corresponded to SSRF postoperative day 27. After his cardiac event, CT revealed hardware failure with one locking screw separating from a plate at the left costochondral junction and another along the sternum (Table 1). The sternal plate had partial separation from the sternum as well (Figure 1). An isolated hardware failure was also noted at the posterolateral segment of a rib close to the transverse process of the corresponding vertebrae. The screw in this instant was not freed to the same extent as was the sternal and costochondral segments and could potentially have been an imaging artifact. The patient ultimately died on HD 56 from PEA arrest.

## Discussion

The overall number of SSRF cases has increased significantly, rising from 1% before 2010 to 10% after 2010 for flail chest injuries [3]. The decision to perform SSRF is often based on clinical judgment; however, flail chest is widely recognized as an indication for surgery with respiratory failure [4]. Correction of flail chest with SSRF results in significantly lower rates of mortality in both the short term (30 days) and long term (2 years) in comparison to nonoperative management [3]. Moreover, SSRF lowers flail chest short-term mortality rates to that of patients with multiple rib fractures and a stable chest wall (no flail chest). Without SSRF, flail chest has a short-term mortality rate that is three times greater than these patients [3]. The assumed conclusion is that the stability of the chest wall, not the number of rib fractures, is the better prognostic indicator. However, the growth in SSRF use has also revealed various surgical complications that deserve consideration.

With any surgical procedure, complications are to be expected. The incidence varies based on the outcome considered, yet hardware failure after SSRF is exceedingly rare. In a recent SSRF systematic review featuring 35 studies and 1,278 patients, only one incident of undefined “mechanical failure” was noted (0.1%) with zero cases of “breakage” (0%) [5]. A separate multicenter study identified 38 cases of hardware failure most frequently in the anterolateral and lateral regions with screw and plate migration as the most commonly cited mechanism [6]. The case we present is therefore rare and further questions SSRF’s effect in patients who later receive CPR and other high impact forces to the anterior chest wall. After reviewing the literature, we have identified only one other case of hardware failure after CPR in an elderly female [1]. She had received identical plates and screws in a similar distribution across the chest wall. The hardware failure in this instance was localized to an anterior plate whose screw had separated from the cortex. Besides this report, we are unaware of any major studies that have considered the impact of CPR on prior SSRF.

## Conclusions

Overall, we suspect that our patient’s changes in fracture pattern with hardware failure were the direct result of chest compressions. The presence of hardware failure in the anterior and anterolateral segments suggests that the sternum and costochondral junction are dynamic areas of the chest wall that dissipate force differently than do the bone of ribs. Future studies should investigate this hypothesis and provide a better understanding of fixation techniques across the entire thoracic cage. The case presented here ultimately questions whether rib fixation systems used beyond the ribs, particularly at the sternum and costochondral junction, ensure ample stability.
